# Olaparib monotherapy for Asian patients with a germline BRCA mutation and HER2-negative metastatic breast cancer: OlympiAD randomized trial subgroup analysis

**DOI:** 10.1038/s41598-020-63033-4

**Published:** 2020-05-29

**Authors:** Seock-Ah Im, Binghe Xu, Wei Li, Mark Robson, Quchang Ouyang, Dah-Cherng Yeh, Hiroji Iwata, Yeon Hee Park, Joo Hyuk Sohn, Ling-Ming Tseng, Carsten Goessl, Wenting Wu, Norikazu Masuda

**Affiliations:** 1 Seoul National University Hospital, Cancer Research Institute, Seoul National University College of Medicine, Seoul, Korea; 2 National Cancer Center/National Clinical Research Center/Cancer Hospital, Chinese Academy of Medical Sciences and Peking Union Medical College, Beijing, China; 3 The First Hospital of Jilin University, Changchun, China; 4 Memorial Sloan Kettering Cancer Center, New York, NY USA; 5 Medical Oncology Center, Hunan Tumor Hospital, Changsha, China; 6 Cheng Ching Hospital, Taichung City, Taiwan; 7 Department of Breast Oncology, Aichi Cancer Center Hospital, Aichi, Japan; 8 Samsung Medical Center, Sungkyunkwan University School of Medicine, Seoul, Korea; 9 Yonsei University College of Medicine, Seoul, Korea; 10 Taipei Veterans General Hospital, National Yang-Ming University, Taipei, Taiwan; 11 AstraZeneca, Gaithersburg, MD USA; 12 National Hospital Organization, Osaka National Hospital, Osaka, Japan

**Keywords:** Randomized controlled trials, Randomized controlled trials, Breast cancer, Breast cancer

## Abstract

The OlympiAD Phase III study (NCT02000622) established the clinical benefits of olaparib tablet monotherapy (300 mg twice daily) over chemotherapy treatment of physician’s choice (TPC) in patients with a germline *BRCA1/2* mutation (gBRCAm) and human epidermal growth factor receptor 2 (HER2)-negative metastatic breast cancer who had received ≤2 chemotherapy lines in the metastatic setting. Here, we report pre-specified analyses of data from Asian (China, Japan, Korea and Taiwan) patients in the study. All patients were randomized 2:1 to olaparib tablets (300 mg twice daily) or single-agent chemotherapy TPC (21-day cycles of either capecitabine, eribulin or vinorelbine). The primary endpoint was progression-free survival assessed by blinded independent central review. The prevalence of gBRCAm in the OlympiAD Asian subgroup screened for study recruitment was 13.5%. Patient demographics and disease characteristics of the Asian subgroup (87/302 patients) were generally well balanced between treatment arms. Asian patients in the olaparib arm achieved longer median progression-free survival, assessed by blinded independent central review, versus the chemotherapy TPC arm (5.7 vs 4.2 months; HR = 0.53 [95% CI: 0.29–0.97]), which was consistent with findings in the global OlympiAD study population. Findings on secondary efficacy and safety/tolerability outcome measures in Asian patients were also similar to those observed in the global OlympiAD study population. The OlympiAD study was not powered to detect race-related differences between treatment groups; however, the consistency of our findings with the global OlympiAD study population suggests that previously reported findings are generalizable to Asian patients.

## Introduction

Over the last few decades there has been a dramatic increase in the incidence of breast cancer (BC), the most prevalent cancer in Asian females^1^, among Asian populations^2,3^. However, the predominance of Western patients in some BC clinical trials raises potential concerns over the generalizability of their findings with respect to effective disease management in routine clinical practice to racially diverse populations, including Asian patients^2,4^.

This is of particular importance because multiple differences exist between Asian and Western patient populations (in their genetic background, socioeconomic status, lifestyle, culture and health beliefs/behaviours) that play an important role in dictating BC incidence and influencing prognosis. For example, with regard to genetic background, Asian patients develop BC at a younger age in comparison to Western patients, therefore the influence of *BRCA1* and *BRCA2* (*BRCA1/2*) mutations in Asian patients are expected to be different than in Western patients^1^. Therefore, BC clinical trials generating data specific to Asian patients with BC are of considerable interest^2,5^.

Olaparib is a potent oral poly(ADP-ribose) polymerase (PARP) inhibitor that has shown clinical efficacy as monotherapy in ovarian, breast and other solid tumour types^6–8^. Previously reported findings from the Phase III OlympiAD trial demonstrated a significant improvement in progression-free survival (PFS) with olaparib tablet monotherapy versus chemotherapy treatment of physician’s choice (TPC) in patients with a germline *BRCA1/2* mutation (gBRCAm) and human epidermal growth factor receptor 2 (HER2)-negative metastatic BC (mBC)^9^. Thus far, outcome measures evaluated in the OlympiAD trial have not been explored to determine the consistency of the clinical benefits of olaparib monotherapy between the global study population (Europe, North America and South America) and a subgroup of Asian patients. To this end, we report pre-specified subgroup analyses to determine the efficacy and safety of olaparib monotherapy versus chemotherapy TPC in the Asian (China, Japan, Korea and Taiwan) subpopulation compared with the global OlympiAD study population.

## Methods

Only a brief overview of the methodology used in the OlympiAD study will be provided here given that full details have been reported previously^9^.

### Study design and patient eligibility

This was a Phase III, multicentre, international, randomized, controlled, open-label study (Clinical trial registration number NCT02000622; 04/12/2013) in adults with BC who met the following key eligibility criteria: (1) triple-negative or HER2-negative and hormone-receptor-positive mBC;(2) deleterious or suspected deleterious gBRCAm that was confirmed via central testing with the BRACAnalysis CDx^®^ test (Myriad Genetics, Inc); (3) received ≤2 previous chemotherapy regimens for metastatic disease, as well as prior anthracycline (unless contraindicated) and a taxane in the adjuvant, neoadjuvant or metastatic setting; (4) if hormone receptor-positive BC, then the patient had received ≥1 endocrine therapy (either adjuvant or metastatic setting) and disease had progressed on therapy, unless the patient was considered ineligible for endocrine treatment. Patients were included regardless of previous platinum-based therapy for BC, but only if their disease had not progressed while receiving that treatment.

Informed consent was provided by all patients who participated in the study and the protocol was approved by the Seoul National University Hospital (EC) Institutional Review Board. The study was performed in accordance with the Declaration of Helsinki, Good Clinical Practice guidelines and the AstraZeneca policy on bioethics.

### Randomization and study treatments

Eligible patients were randomized using an interactive voice or Web response system at centres in China, Japan, Korea and Taiwan in a 2:1 ratio to either olaparib monotherapy (300 mg twice daily [bid]; tablets) or predeclared single-agent chemotherapy TPC (capecitabine, eribulin or vinorelbine [21-day cycles]). In China, only capecitabine or vinorelbine were considered in the chemotherapy TPC arm because eribulin did not have regulatory approval at that time. Study treatment was continued until patients’ disease progressed or unacceptable side effects were experienced. Crossover from chemotherapy TPC to olaparib within the study was not allowed.

### Study endpoints and assessments

Findings on the primary endpoint, PFS by blinded independent central review (BICR; modified Response Evaluation Criteria in Solid Tumors version 1.1 criteria), and secondary endpoints in the OlympiAD study have been reported previously^9^. Secondary endpoints included time to second progression or death (PFS2), overall survival (OS), objective response rate (ORR), and safety and tolerability. Computed tomography or magnetic resonance imaging were used to assess tumours. Reported adverse events (AEs) were graded using the National Cancer Institute’s Common Terminology Criteria for Adverse Events version 4.0.

### Statistical analysis

To consider consistency of treatment effect across potential or expected prognostic factors, the OlympiAD study design incorporated several predefined subgroup analyses, including geographical region (Asia, Europe, North America and South America). The Asian subgroup included patients from China, Japan, Korea and Taiwan.

Efficacy analyses were conducted using the full analysis set, whereas the safety analysis set included all patients who had received ≥1 dose of study treatment. The Kaplan–Meier method was used to generate time-to-event curves, from which medians were calculated. For analyses of survival (PFS, PFS2 and OS) in Asian patients, the hazard ratios (HRs) and associated 95% confidence intervals (CIs) were estimated using an unstratified log-rank test. A HR of <1 favoured olaparib 300 mg bid over chemotherapy TPC.

### Ethical approval/informed consent

The institutional review boards or independent ethics committees of all investigational sites approved the protocol. The study was performed in accordance with the Declaration of Helsinki, Good Clinical Practice, and the AstraZeneca policy on bioethics^10^. Informed consent was provided by all patients who participated in the study and the protocol was approved by the Seoul National University Hospital (EC) Institutional Review Board and the ethics review committees at the participating institutions.

## Results

### Asian patients in the OlympiAD study

Among patients with unknown BRCA status who were screened for the OlympiAD study using a central Myriad test, the prevalence of a deleterious or suspected deleterious BRCA mutation was 13.5% (121/895) in Asian patients (*BRCA1*, 54/121 [44.6%]; *BRCA2* 64/121 [52.9%]; both *BRCA1* and *BRCA2* 3/121 [2.5%]) compared with 11.4% (101/888; *BRCA1*, 58/101 [57.4%]; *BRCA2* 43/101 [42.6%]) in Caucasian patients, and 10.4% (14/135; *BRCA1*, 8/14 [57.1%]; *BRCA2* 6/14 [42.9%]) in ‘other’ patients.

A total of 302 patients between 7 April 2014 and 27 November 2015 were randomized to olaparib 300 mg bid tablet monotherapy (n = 205) or chemotherapy TPC (n = 97), which included a subgroup of 87 Asian patients (Supplementary Fig. 1). Within the Asian subgroup, 59 and 28 patients were allocated to the olaparib and chemotherapy TPC arms, respectively. One Asian patient declined study treatment because of their allocation to the chemotherapy TPC arm. Patient demographics and disease characteristics of the Asian subgroup were generally well balanced between treatment arms (Table 1). Each of the treatment arms comprised patients from China, Japan, Korea and Taiwan; among olaparib recipients, 32 (54.2%) patients were from China, 15 (25.4%) were from Japan, 11 (18.6%) were from Korea, and one (1.7%) was from Taiwan, and for patients receiving chemotherapy TPC, nine (32.1%) patients each were from China, Japan and Korea, and one patient (3.6%) was from Taiwan.

**Table 1:** Patient demographics and disease characteristics of the Asian subgroup and the global OlympiAD study population.

	**Asian subgroup**		**Global OlympiAD study population**	
	**Olaparib** **(N** = **59)**	**Chemotherapy TPC** **(N** = **28)**	**Olaparib** **(N** = **205)**	**Chemotherapy TPC** **(N** = **97)**
Median age, years (range)	46.0 (28–74)	47.0 (24–66)	44.0 (22–76)	45.0 (24–68)
Median body weight, kg (range)	59.0 (36–85)	55.5 (39–86)	64.0 (36–113)	62.0 (39–112)
Male, n (%)	1 (1.7)	1 (3.6)	5 (2.4)	2 (2.1)
**ECOG performance status, n (%)**				
0 1	42 (71.2) 17 (28.8)	20 (71.4) 8 (28.6)	148 (72.2) 57 (27.8)	62 (63.9) 35 (36.1)
BRCAm status, n (%)*				
*BRCA1* *BRCA2* Both Missing	30 (52.8) 25 (42.4) 3 (5.1) 1 (1.7)	12 (42.9) 16 (57.1) 0 0	117 (57.1) 84 (41.0) 4 (2.0) 0	51 (52.6) 46 (47.4) 0 0
**Hormone receptor status, n (%)**				
ER+ and/or PR+ TNBC	29 (49.2) 30 (50.8)	13 (46.4) 15 (53.6)	103 (50.2) 102 (49.8)	49 (50.5) 48 (49.5)
Prior chemotherapy for mBC, n (%)	41 (69.5)	23 (82.1)	146 (71.2)	69 (71.1)
Prior platinum treatment, n (%)	21 (35.6)	8 (28.6)	60 (29.3)	26 (26.8)
Chemotherapy TPC, n (%)^†^	NA		NA	
Capecitabine Vinorelbine Eribulin		10 (35.7) 5 (17.9) 12 (42.9)		41 (42.3) 16 (16.5) 34 (35.1)

Data cut-off 9 December 2016; *BRCA mutation confirmed using BRACAnalysis CDx^®^ (Myriad Genetic Laboratories, Inc); ^†^Six patients did not receive study treatment in the global population, one of whom was in the Asian subgroup. No patients in China received eribulin as chemotherapy TPC because it did not have regulatory approval in that country. BRCAm, BRCA mutation; ECOG, Eastern Cooperative Oncology Group; ER+, oestrogen receptor positive; NA, not applicable; PR+, progesterone receptor positive; TNBC, triple-negative breast cancer; TPC, treatment of physician’s choice.

### Efficacy

#### PFS

**Figure 1:**
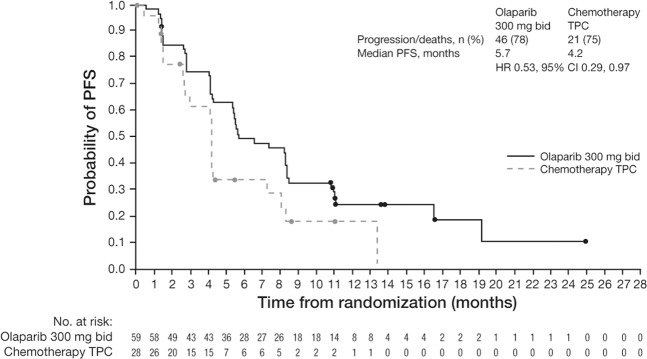
PFS (as determined by BICR) in the olaparib and chemotherapy TPC arms in the Asian subgroup and global OlympiAD study population. Data cut-off 9 December 2016; PFS data maturity, 77% (67/87 patients). BICR, blinded independent central review; bid, twice daily; CI, confidence interval; HR, hazard ratio; PFS, progression-free survival; TPC, treatment of physician’s choice.

**Figure 2:**
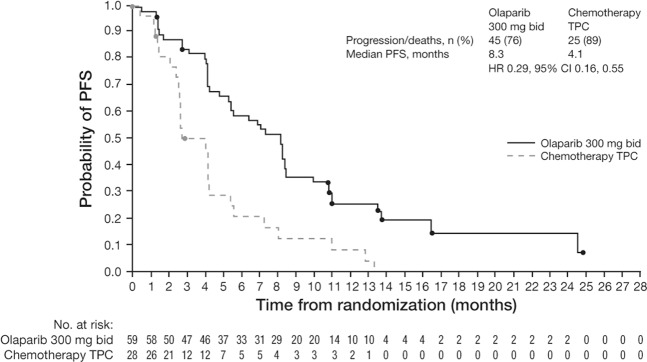
PFS (as determined by investigator assessment) in the olaparib and chemotherapy TPC arms in the Asian subgroup. Data cut-off 9 December 2016; PFS data maturity, 80% (70/87 patients). Median time from randomization to censoring (censored patients only): 11.1 months olaparib and 4.4 months chemotherapy. bid, twice daily; CI, confidence interval; HR, hazard ratio; PFS, progression-free survival; TPC, treatment of physician’s choice.

Asian patients in the olaparib arm achieved longer median PFS compared with the chemotherapy TPC arm (5.7 vs 4.2 months; HR = 0.53 [95% CI: 0.29–0.97]; data maturity 77%), as assessed by BICR (Fig. 1). This BICR-assessed PFS benefit of olaparib over chemotherapy TPC in the Asian subgroup was consistent with that observed in the global OlympiAD study population (7.0 vs 4.2 months; HR = 0.58 [95% CI: 0.43–0.80])^9^. Furthermore, the improvement in BICR-assessed PFS associated with olaparib was supported by investigator-assessed PFS (8.3 vs 4.1 months; HR = 0.29 [95% CI: 0.16–0.55]; data maturity 80%) (Fig. 2).

#### PFS2, OS and ORR

**Figure 3:**
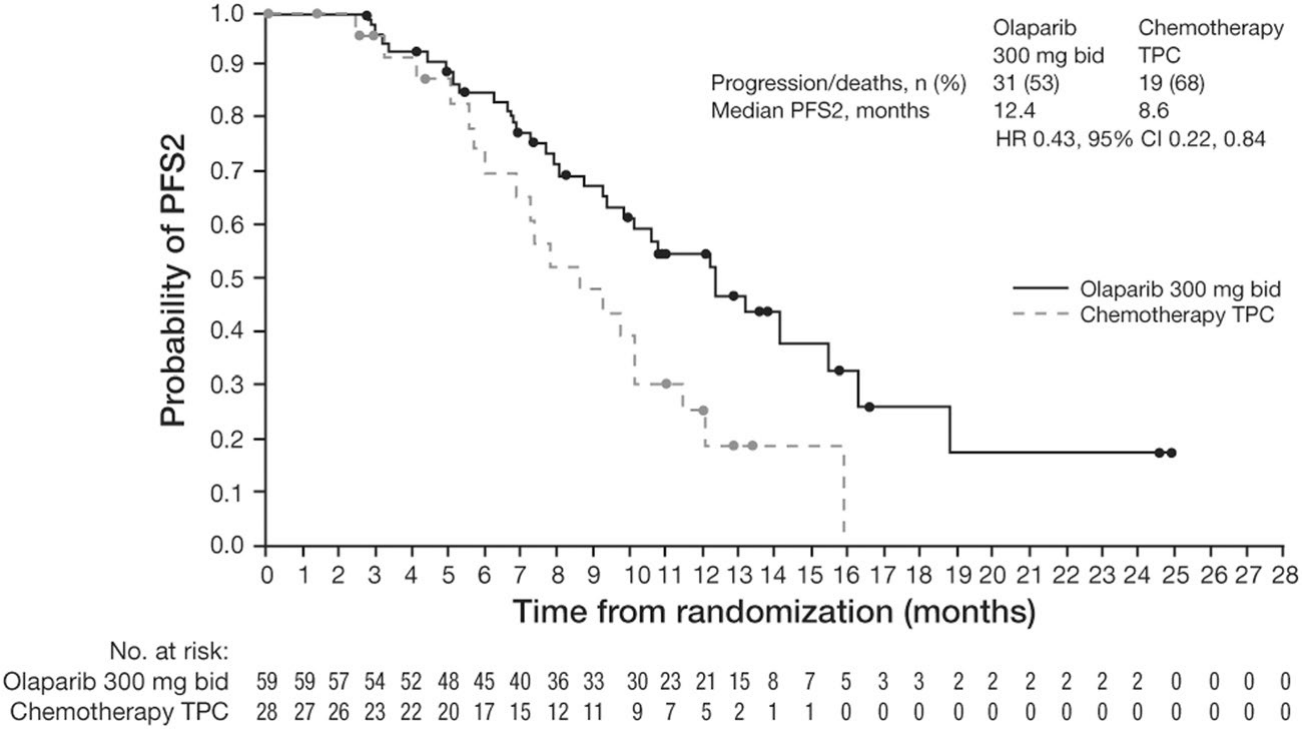
PFS2 (as determined by investigator assessment) in the olaparib and chemotherapy TPC arms in the Asian subgroup. Data cut-off 9 December 2016; PFS2 data maturity, 57% (50/87 patients). bid, twice daily; CI, confidence interval; HR, hazard ratio; PFS2, time to second progression or death; TPC, treatment of physician’s choice.

**Figure 4:**
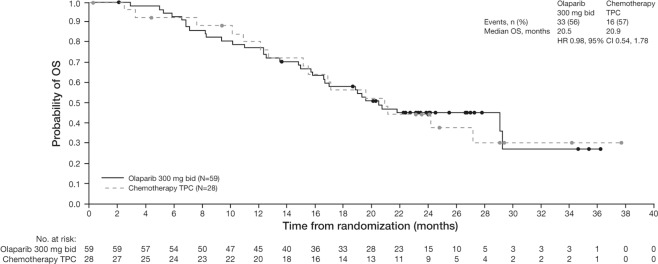
OS in the olaparib and chemotherapy TPC arms in the Asian subgroup. Data cut-off 25 September 2017; OS data maturity, 56% (34/87 patients). bid, twice daily; CI, confidence interval; HR, hazard ratio; OS, global survival; TPC, treatment of physician’s choice.

Asian patients in the olaparib arm also achieved a longer median time to PFS2 compared with chemotherapy TPC (12.4 vs 8.6 months; HR = 0.43 [95% CI: 0.22–0.84]; data maturity 57%) (Fig. 3). In the Asian subgroup analysis, at the time of the data cut-off for the final OS analysis, median OS was similar in the olaparib and chemotherapy TPC arms (20.5 vs 20.9 months; HR = 0.98 [95% CI: 0.54–1.78]; data maturity 56.3%; median follow-up was 19.3 and 18.4 months for the olaparib and chemotherapy TPC arms, respectively) (Fig. 4).

ORR was higher for evaluable Asian patients in the olaparib arm (63.6% [95% CI: 47.8–77.6]) than in the chemotherapy TPC arm (38.1% [95% CI: 18.1–61.6]). Higher rates of BICR-assessed complete response and partial response were reported among Asian patients in the olaparib arm versus the chemotherapy TPC arm (1.7% vs 0% and 47.5% vs 28.6%, respectively). A best objective response of stable disease lasting ≥11 weeks was achieved by 30.5% and 25.0% of Asian patients in the olaparib and chemotherapy TPC arms, respectively.

Moreover, Asian patients showed a longer median (interquartile range) duration of response with olaparib versus chemotherapy TPC (4.2 [2.8–8.2] vs 2.8 months [2.1–12.2]). In the Asian subgroup, median (interquartile range) time to onset of response was similar in each treatment arm (olaparib, 1.5 months [1.4–2.8]; chemotherapy TPC, 1.5 months [1.3–2.8]).

### Safety and tolerability

Median (range) duration of treatment in OlympiAD Asian patients was longer in the olaparib arm compared with the chemotherapy TPC arm (253 [14–1103] vs 91 [21–385] days). Median relative dose intensity in the Asian subgroup was 99.4% and 93.6% in the olaparib and chemotherapy TPC arms, respectively.

The safety and tolerability profile of olaparib in Asian patients was broadly consistent with that reported previously in the global OlympiAD study population (Table 2). In the olaparib arm, nausea and anaemia were the most commonly reported AEs among Asian patients (54.2% and 44.1%, respectively) and the global OlympiAD study population (58.0% and 40.0%, respectively). AEs reported more frequently in the olaparib arm by Asian patients than in the global OlympiAD study population (>10% difference between subgroup and global population) were decreased white blood cell (WBC) count (39.0% vs 16.1%, respectively) and elevated transaminase levels (increased alanine aminotransferase, 27.1% vs 11.7%; increased aspartate aminotransferase, 22.0% vs 9.8%). In the olaparib arm, AEs reported less frequently by Asian patients versus the global OlympiAD study population (>10% difference between subgroup and global population) were neutropenia (8.5% vs 27.3%) and fatigue (16.9% vs 29.8%). Diarrhoea in the olaparib arm was reported by 15.3% and 20.5% of Asian patients and the global OlympiAD study population, respectively.

**Table 2:** Summary of AEs in the Asian subgroup and the global OlympiAD study population.

	Asian subgroup				Global OlympiAD study population			
	Olaparib (N = 59)		Chemotherapy TPC (N = 27)		Olaparib (N = 205)		Chemotherapy TPC (N = 91)	
	Any grade	Grade ≥3	Any grade	Grade ≥3	Any grade	Grade ≥3	Any grade	Grade ≥3
Any AE	59 (100)	27 (45.8)	26 (96.3)	16 (59.3)	200 (97.6)	78 (38)	87 (95.6)	45 (49.5)
Nausea	32 (54.2)	0	11 (40.7)	0	119 (58.0)	0	32 (35.2)	1 (1.1)
Anaemia^†^	26 (44.1)	12 (20.3)	9 (33.3)	4 (14.8)	82 (40.0)	33 (16.1)	24 (26.4)	4 (4.4)
Neutropenia^‡^	5 (8.5)	1 (1.7)	7 (25.9)	4 (14.8)	56 (27.3)	19 (9.3)	45 (49.5)	24 (26.4)
Decreased WBC count	23 (39.0)	6 (10.2)	14 (51.9)	8 (29.6)	33 (16.1)	7 (3.4)	19 (20.9)	9 (9.9)
Vomiting	17 (28.8)	0	6 (22.2)	0	66 (32.2)	0	14 (15.4)	1 (1.1)
Increased ALT	16 (27.1)	1 (1.7)	7 (25.9)	1 (3.7)	24 (11.7)	3 (1.5)	16 (17.6)	1 (1.1)
Increased AST	13 (22.0)	2 (3.4)	8 (29.6)	0	20 (9.8)	5 (2.4)	15 (16.5)	0
Decreased appetite	11 (18.6)	0	7 (25.9)	0	35 (17.1)	0	11 (12.1)	0
Upper respiratory tract infection	10 (16.9)	1 (1.7)	6 (22.2)	0	27 (13.2)	1 (0.5)	9 (9.9)	0
Fatigue	10 (16.9)	0	1 (3.7)	0	61 (29.8)	7 (3.4)	22 (24.2)	1 (1.1)
Diarrhoea	9 (15.3)	0	6 (22.2)	0	42 (20.5)	1 (0.5)	20 (22.0)	0
Pyrexia	8 (13.6)	0	6 (22.2)	0	30 (14.6)	0	16 (17.6)	0
Headache	7 (11.9)	0	4 (14.8)	1 (3.7)	42 (20.5)	2 (1.0)	14 (15.4)	2 (2.2)
PPE syndrome	1 (1.7)	0	5 (18.5)	1 (3.7)	1 (0.5)	0	19 (20.9)	2 (2.2)
Dose reduction due to AEs, n (%)	10 (16.9)	—	8 (29.6)	—	52 (25.4)	—	28 (30.8)	—
Treatment interruption due to AEs, n (%)	17 (28.8)	—	6 (22.2)	—	74 (36.1)	—	26 (28.6)	—
Treatment discontinuations because of AEs, n (%)	4 (6.8)	—	2 (7.4)	—	10 (4.9)	—	7 (7.7)	—

Most common AE of any grade occurring in ≥20% of Asian patients or the global OlympiAD study population in either treatment arm. AEs were graded using the National Cancer Institute’s Common Terminology Criteria for Adverse Events version 4.0, and data were collected for the duration of study and the 30-day post-treatment follow-up period. Data cut-off 25 September 2017; ^†^Anaemia includes anaemia, decreased haemoglobin level, decreased haematocrit, decreased red blood cell count and erythropenia; ^‡^Neutropenia includes febrile neutropenia, granulocytopenia, decreased granulocyte count, neutropenia, neutropenic sepsis, decreased neutrophil count and neutropenic infection. AE, adverse event; ALT, alanine aminotransferase; AST, aspartate aminotransferase; PPE, palmar–plantar erythrodysesthesia; WBC, white blood cell.

The incidence of grade ≥3 AEs in Asian patients (olaparib, 45.8%; chemotherapy TPC, 59.3%) was similar to that in the global OlympiAD study population (olaparib, 38.0%; chemotherapy TPC, 49.5%) (Table 2). The most commonly reported grade ≥3 AE in the olaparib arm among Asian patients and the global OlympiAD study population was anaemia (20.3% and 16.1%, respectively). A total of 8 (13.6%) and 2 (7.4%) Asian patients receiving olaparib or chemotherapy TPC, respectively, received blood transfusions and in the global OlympiAD study population, 37 (18.0%) and 5 (5.5%) patients received blood transfusions, respectively. In the olaparib arm, grade ≥3 AEs of decreased WBC count were more commonly reported in Asian patients than in the global OlympiAD study population (10.2% vs 3.4%), whereas grade ≥3 neutropenia was less commonly reported (1.7% vs 9.3%).

In the olaparib arm, the proportions of Asian patients who experienced dose reduction, treatment interruption/delay, or treatment discontinuation due to AEs (16.9%, 28.8%, and 6.8%, respectively) were similar to those in the global OlympiAD study population (25.4%, 36.1%, and 4.9%, respectively) (Table 2). AEs leading to treatment discontinuation among Asian patients in the olaparib arm included anaemia (n = 3), increased intracranial pressure (n = 1), and decreased platelet count (n = 1). In the chemotherapy TPC arm, AEs of anaemia (n = 1) and decreased neutrophil count (n = 1) led to treatment discontinuation in Asian patients. There were no incidences of acute myeloid leukaemia, myelodysplastic syndromes, or pneumonitis reported in Asian patients or the global OlympiAD study population in either treatment arm (one patient in the global OlympiAD study population reported an AE of pneumonitis which was subsequently confirmed to be misdiagnosed as pneumonitis and was actually related to disease progression). No Asian patient died as the result of an AE in either treatment arm of the OlympiAD study.

## Discussion

Approximately 29% of patients in the global OlympiAD study population were Asian. The prevalence of gBRCAm in Asian patients screened for recruitment into the OlympiAD study (13.5%) is consistent with that across other studies of Asian patients with BC (familial BC, 8.0–31.8%; early-onset BC, 2.8–21.4%)^1^. These rates are high and warrant increased awareness of, and screening for, *BRCA1/2* mutations among Asian populations. Notably, researchers conducting a whole-exome and whole-transcriptome profiling study identified not only higher frequencies of *BRCA1/2* mutation in younger Korean patients with BC tumours, but also enrichment of a mutation signature linked to homologous recombination repair (HRR) deficiency in triple-negative BC (TNBC)^11^. Their findings suggested that PARP inhibitors may prove to be a particularly potent therapeutic intervention among younger Asian patients with TNBC.

In general, the baseline characteristics (age, gender, ECOG performance status and hormone receptor status) were generally well balanced between treatment arms and between the Asian subgroup and the global population. In the Asian subgroup, fewer patients receiving olaparib had received prior chemotherapy for mBC compared with those receiving chemotherapy TPC (70 vs 82%). The *BRCA1/2* mutation status was also slightly different in the Asian subgroup between treatment groups with more patients receiving olaparib treatment harbouring a *BRCA1* mutation (53 vs 43% for chemotherapy TPC), and conversely more patients receiving chemotherapy TPC had a *BRCA2* mutation (57 vs 42% for olaparib); subgroup analyses of PFS in the global OlympiAD study population showed that the type of BRCA mutation did not appear to influence the benefit of olaparib treatment^9^. When compared with the global study population, Asian patients in both treatment arms had a lower median body weight, and a greater number of Asian patients in the chemotherapy TPC arm had received prior chemotherapy for metastatic disease (82%) compared with the global population (71%). There was also a difference between the Asian and global populations in the type of chemotherapy patients received during the study with Asian patients more likely to receive eribulin (43%) than the global population (35%), whereas in the global population the most common type of chemotherapy received was capecitabine (42% vs 36% for Asian patients).

Findings from our pre-specified analyses suggested that the BICR-assessed PFS benefit achieved by Asian patients in the olaparib arm compared with those in the chemotherapy TPC arm was consistent with the previously reported primary endpoint in the global OlympiAD study population^9^. This effect was further substantiated by the investigator-assessed PFS benefit of olaparib over chemotherapy TPC in the Asian subgroup, which was, again, consistent with that observed in the global OlympiAD study population (7.8 vs 3.8 months; HR = 0.50 [95% CI: 0.36–0.68]; *P* < 0.001)^9^. The slight imbalances in the baseline characteristics between the Asian subgroup treatment arms and between the Asian and global populations do not appear to have had any meaningful effect on the BICR or investigator-assessed PFS results.

The maintenance of benefit beyond first progression for olaparib compared with chemotherapy TPC was supported by PFS2 findings in the Asian subgroup that reflected those observed in the global OlympiAD study population (13.2 vs 9.3 months; HR = 0.57 [95% CI: 0.40–0.83]; *P* = 0.003)^9^. The continued benefit of olaparib following initial progression demonstrates that olaparib does not affect the efficacy of subsequent treatments. Antitumour activity findings in Asian patients suggested an advantage of olaparib over chemotherapy TPC that was consistent with that reported in the global OlympiAD study population in terms of not only ORR (59.9% [95% CI: 52.0–67.4] vs 28.8% [95% CI:18.3–41.3]), but also median duration of response (6.4 [2.8–9.7] vs 7.1 months [3.2–12.2])^9^. Furthermore, in line with the global OlympiAD study population, there was no delay in the time to response for Asian patients receiving olaparib compared with those receiving chemotherapy TPC with the median time to onset of response being 1.5 months in both arms; this finding is an important consideration for symptomatic or rapidly progressing patients.

Our results showed that the safety and tolerability profile of olaparib in Asian patients was generally similar to that reported previously in the global OlympiAD study population^9^, suggesting that the slight imbalances in the baseline characteristics between treatment arms for the Asian subgroups has not impacted the safety of olaparib in these patients. Asian patients had a longer median duration of treatment in the olaparib arm compared with the chemotherapy TPC arm with a high relative dose intensity (olaparib arm, 99.4%; chemotherapy TPC, 93.6%). It has been proposed that there may be race-related differences in tolerability and response to anticancer drugs in patients with BC^12^. For example, findings from a retrospective database review of patients with early-stage BC suggested that Asian patients receiving adjuvant doublet therapy (docetaxel plus cyclophosphamide) had a higher liability for grade ≥3 neutropenia compared with their Western counterparts (>30% vs 4%)^13^. Our data showed less marked contrasts in incidences of grade ≥3 AEs between Asian patients and the global OlympiAD study population. In the olaparib arm, the greatest contrasts in incidence of grade ≥3 AEs between Asian patients and the global OlympiAD study population were for decreased WBC count (10.2% vs 3.4%), although neutropenia was not more frequent in Asian patients (1.7% vs 9.3% in the global population). Additionally, there were no reports of febrile neutropenia in either Asian patients or those from the global OlympiAD study population receiving olaparib.

The finding of similar efficacy and safety in Asian and global study populations was not unexpected as the pharmacokinetics of olaparib tablets have been reported to be similar between Asian and Western patients^14^. The consistency of findings supports similar clinical benefits of olaparib tablet monotherapy over chemotherapy TPC in patients with a gBRCAm and HER2-negative mBC in Asian patients and the global OlympiAD study population.

A review addressing future needs for Asian BC research prioritized diagnostic and prognostic studies and called for the recruitment of patients from various Asian settings into international clinical trials to advance understanding of the effectiveness of novel therapeutic interventions in this subpopulation^2^. The OlympiAD study is a step towards addressing this need by recruiting a substantial proportion of Asian patients (~29%).

## Conclusions

Findings from these pre-specified analyses demonstrated a greater clinical benefit in Asian patients for olaparib tablet monotherapy compared with chemotherapy TPC. This clinical benefit observed in Asian patients is consistent with that reported in the global OlympiAD study population, as measured by BICR-assessed PFS (the primary endpoint) and investigator-assessed PFS, PFS2 and ORR. Olaparib was generally well tolerated in Asian patients and was characterized by low discontinuation rates as a result of toxicity, as well as a lower rate of grade ≥3 AEs compared with chemotherapy TPC. The safety profile of olaparib in the Asian subpopulation was broadly consistent with that observed in the global OlympiAD study population.

## Supplementary information

Supplementary information.
