# Supplementary material for: Olaparib monotherapy for Asian patients with a germline BRCA mutation and HER2-negative metastatic breast cancer: OlympiAD randomized trial subgroup analysis
Source: Sci Rep. 2020 May 29;10:8753. doi: 10.1038/s41598-020-63033-4 (PMC7260217; doi:10.1038/s41598-020-63033-4)

SUPPLEMENTARY APPENDIX

Olaparib monotherapy for Asian patients with a germline BRCA mutation and HER2-negative metastatic breast cancer: OlympiAD randomized trial subgroup analysis

Seock-Ah Im, Binghe Xu, Wei Li, Mark Robson, Quchang Ouyang, Dah-Cherng Yeh, Hiroji Iwata, Yeon Hee Park, Joo Hyuk Sohn, Ling-Ming Tseng, Carsten Goessl, Wenting Wu, Norikazu Masuda

Supplementary Fig. 1 Patient flow diagram

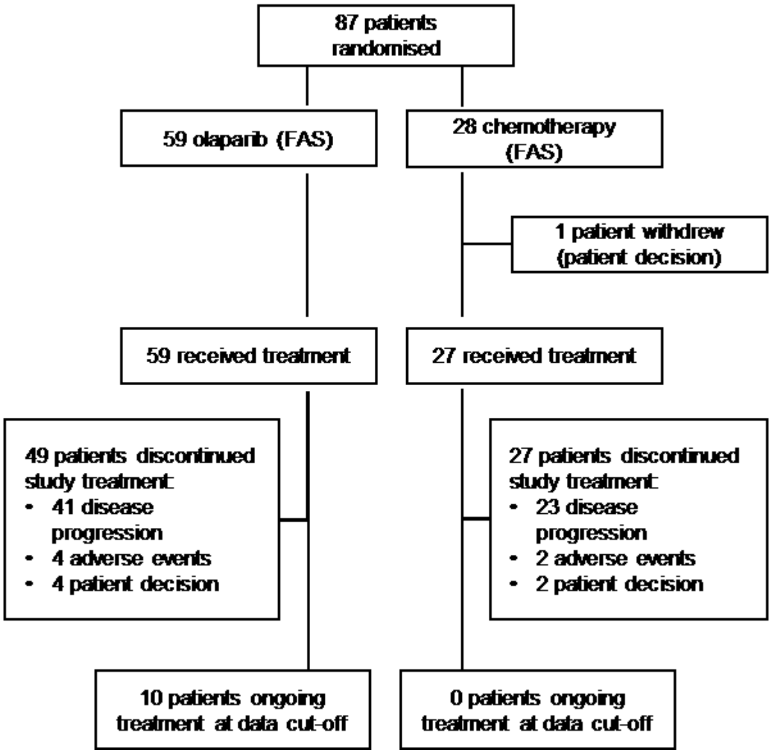

Supplement: Supplementary file 1 — Supplementary information. [file 41598_2020_63033_MOESM1_ESM.pdf]
